# Differences in Vertical Jump Force-Time Characteristics between Stronger and Weaker Adolescent Basketball Players

**DOI:** 10.3390/sports5030063

**Published:** 2017-08-24

**Authors:** Christopher Thomas, Irene Kyriakidou, Thomas Dos’Santos, Paul A. Jones

**Affiliations:** 1 Directorate of Sport, Exercise and Physiotherapy, University of Salford, Salford M6 6PU, UK; t.dossantos@hotmail.co.uk (T.D.); p.a.jones@salford.ac.uk (P.A.J.); 2 Research Institute of Sport and Exercise Sciences, Liverpool John Moores University, Liverpool L3 5UA, UK; I.Kyriakiou@2016.ljmu.ac.uk

**Keywords:** maximum strength, reactive strength, isometric mid-thigh pull, countermovement jump, drop jump

## Abstract

The countermovement jump (CMJ) and isometric mid-thigh pull (IMTP) are commonly used to compare one’s force capacity during dynamic and isometric assessments, respectively. However, little research has investigated the influence of maximum isometric strength on drop-jump (DJ) performance. Therefore, the purpose of this study was to explore differences in CMJ and DJ force-time characteristics between stronger and weaker adolescent male basketball players. Sixteen adolescent male basketball players performed the IMTP to assess measures of peak force (IMTP PF), whereas CMJ and DJ calculated a range of kinetic and kinematic variables. Peak concentric force (CMJ-PF) in the CMJ was greater for stronger players (*d* = 1.99). However, no differences in DJ force-time characteristics existed between stronger and weaker players. Future research should be undertaken to investigate the role of maximum strength on DJ force-time characteristics in adolescent male basketball players. Such studies may help direct the creation of athlete training and monitoring programs more effectively to represent accurate player profiling.

## 1. Introduction

Previous literature indicates that isometric measures of maximum strength have strong correlations with vertical jump [squat jump (SJ) and countermovement jump (CMJ)] performances [1,2,3]. Basketball players must successfully complete multiple high-intensity short-duration sprints, cutting and pivoting manoeuvres, in addition to 40–60 jumps and landings [4] during competition, all of which require high levels of reactive-strength and force-generating capabilities. Thus, it is no surprise that numerous studies have attempted to provide normative data on such physical attributes in male [5,6,7,8,9] and female [10,11] basketball players. Given that basketball players repeatedly perform sprinting, drop jumps (DJ), bounding and rebounding, it seems pertinent to investigate this topic within this cohort.

The ability of basketball athletes to effectively sprint, turn, change direction, jump and land is highly related to an athlete’s maximum strength [12]. Previous research has reported measures of maximum strength to be strongly associated with superior vertical jump (*r* = 0.64–0.74) [7,9], horizontal jump (*r* = 0.67) [9], sprint (*r* = 0.63–0.65) [6] and change of direction (*r* = 0.79–0.89) [10] performances, regardless of sex. Furthermore, maximum strength has shown to strongly correlate (*r* = 0.74) with total playing time in male collegiate basketball players [7], has been able to distinguish between high- and lower-level competitors [5], and demonstrate small to large associations with CMJ reactive strength index-modified (CMJ-RSImod) [13]. However, the majority of research has focused on ‘global’ measures of performance in general, without considering the specific kinetic and kinematic variables which may have a role during these dynamic tasks. Furthermore, there is a lack of research investigating the role of maximum strength in tasks which require high levels of reactive-strength (DJ) [14,15,16,17].

Reactive-strength can be defined as the ability to utilise the stretch-shorten cycle (SSC), whereby a force concentric muscle action is preceded by a rapid eccentric muscle action [18]. Stretch-shortening cycle movements can be classified as fast (<250 ms: sprinting, DJ, bounding) and slow (>250 ms: depth-jumps, CMJ, change of direction) [19], thus it is important for athletes to use a variety of drills to enhance reactive-strength performance [20]. The DJ, performed either from a standardized height or a variety of heights, is commonly used both as part of athlete training programs to promote the development of lower body power [17,21] and athlete testing batteries to provide insight into strength diagnostics [14,22]. Many studies calculate ‘reactive-strength index’ (DJ-RSI) during DJ assessment by the equation: DJ-RSI = jump height/ground contact time, and as previously mentioned, this can be implemented over a series of box heights to identify an athlete’s reactive-strength over different eccentric stretch loads [14,15,16]. A major limitation of these studies is that they only examine ‘global’ measures of DJ performance (jump height, ground contact time, and resultant DJ-RSI) [14], thus only providing insight into a small part of the DJ performance. Previous evidence questions the use of RSI as a measurement of DJ performance [15], and that the addition of other kinetic and kinematic variables [15,16] could provide a more comprehensive understanding of the reported factors thought to underpin superior DJ performance to create an accurate player profile.

Early research suggests maximum strength, specifically isometric mid-thigh pull peak force (IMTP PF) to demonstrate moderate to very strong relationships with slow SSC tasks (CMJ) variables (*r* = 0.53–0.82), such as PF, peak power, and jump height in a range of populations such as weightlifting [23], cycling [3], recreationally trained [24], wrestling [25], American football [2], and soccer [26] because of similarities in vertically directed force production and the subsequent biomechanically derived variables. Apart from a few studies [14,27], much less is known about the influence of maximum strength on reactive-strength measures. A recent study [14] found that stronger athletes had larger DJ-RSI at drop heights of 0.4 m (*p* = 0.027; *d* = 1.02), 0.5 m (*p* = 0.010; *d* = 1.21) and 0.6 m (*p* = 0.004; *d* = 1.39) but not at 0.3 m (*p* = 0.062; *d* = 0.84) when compared to weaker athletes. Also, stronger athletes were able to maintain their reactive-strength ability as box heights increased from 0.3 to 0.6 m, indicating the importance of relative maximal strength to overcome high eccentric loads. One limitation of this study was the use of photo-electric cells to assess DJ performance, which although practical in terms of setup, data collection, and data analysis, has shown to affect JH by approximately 6% [28]. In another study, stronger rugby players (one repetition maximum (1RM) back squat ≥ 1.9 × body mass) showed significantly higher DJ-RSI at higher box heights (0.5 m) compared to weaker athletes (1RM back squat ≤ 1.5 × body mass) [27]. Moreover, the authors performed DJ on a force platform, thus allowing more insight into the biomechanical principles underpinning DJ performance. However, Dymond et al. [27] failed to include the components which make up DJ-RSI; jump height and ground contact time; furthermore, additional kinetic and kinematic variables could have been included in this study to provide a more comprehensive insight into DJ performance. The aim of this study, therefore, was to explore differences in CMJ and DJ force-time characteristics between stronger and weaker adolescent male basketball players. It was hypothesized that all force-time variables obtained in CMJ and DJ would be higher for stronger than weaker athletes.

## 2. Materials and Methods

### 2.1. Subjects

Sixteen adolescent male basketball players (age 17.3 ± 0.6 years; height 186.1 ± 9.8 cm; mass 80.7 ± 10.0 kg) participated in this study. Subjects attended a single testing session scheduled at the same time of day in a laboratory setting. Testing was conducted in the preseason during which time all subjects were training with sessions comprising all the elements of basketball performance including 4–5 basketball specific training session, plus two resistance training sessions each week. Testing took place at 10:00–12:00 in place of a normal skills training session. Subjects who suffered from a previous anterior cruciate ligament injury were excluded from the study. Likewise, subjects who suffered from any other lower limb injury prior within the 6 months prior to data collection were subsequently excluded. The investigation was approved by the institutional review board, and all provided appropriate consent to participate, with consent from the parent or guardian of all players under the age of 18. The study conformed to the principles of the World Medical Association’s Declaration of Helsinki.

### 2.2. Procedures

A cross sectional design was used to investigate the influence of maximum isometric strength on CMJ and DJ force-time characteristics in adolescent male basketball players. On arrival, all subjects had their height (Stadiometer, Model 213, Seca, Birmingham, UK) and body mass assessed (Seca Digital Scales, Model 707, Seca, Birmingham, UK) while in bare feet, measured to the nearest 0.1 and 0.1 cm, respectively. Subjects were required to abstain from training for 48 h before testing and asked to maintain a consistent fluid and dietary intake on the day of testing as they would for normal skills training. Testing order was as follows: CMJ, DJ and IMTP. Before the start of testing, athletes performed a standardized warm-up of activation and mobilization exercises, including various bodyweight lunges and squats, followed by some low level plyometric drills, replicating the participant’s standardized warm-ups before training. Furthermore, standardized progressive warm-ups were applied before all tests to control potential variables and improve the reliability of all tests.

### 2.3. Vertical Jumps

Vertical jump assessments included CMJ and DJ. For the CMJ, subjects were instructed to jump “as high and as fast as possible”. Countermovement jumps were performed with the hands on the hips, and countermovement depth of the eccentric phase was self-selected by the subjects to maximize CMJ height and ecological validity. Subjects performed three trials, with one minute of rest between trials.

For the DJ, subjects were instructed to “step out” from a 0.3 m box, with the lead leg with their hands on the hips, and not jump from the box, to ensure a homogeneous drop distance on each trial. Further, subjects were requested to jump for maximum height and minimum contact time with the force platform via the instruction “jump as high and as fast as possible” [29]. Subjects performed three trials, with one minute of rest between trials.

Countermovement jump and DJ data were collected using a portable force platform sampling at 1000 Hz (Kistler Instrument Corporation, Winterthur, Switzerland, Model 9286AA, SN 1209740). The force platform was interfaced with a laptop to allow for direct measurement of force-time characteristics, and then analysed using Bioware software (Version 5.11; Kistler Instrument Corporation, Winterthur, Switzerland) and applied to a customised analysis spreadsheet. Prior to the onset of the countermovement, subjects remained stationary on the force platform for one second to enable an accurate measurement of body weight. Vertical ground reaction force data were then averaged across the first second, and the onset of the countermovement was determined when this value was reduced by 5 SDs [30]. Countermovement jump time to take-off was calculated from the force-time record as the length of time between the onset of the countermovement and the point of take-off [31]. Take-off and landing was identified as the point when the vertical ground reaction force descended and ascended past 20 N, respectively. Reactive strength index-modified (RSImod) was calculated by dividing jump height by the time to take-off. Concentric peak force (PF) was determined from the unfiltered force–time history [32] and was presented as a value relative to body mass (N·kg^−^¹). Jump height (JH) was calculated based on the vertical velocity at take-off [33]. Peak concentric power (PP) was then taken as the product of the vertical velocity and vertical ground reaction force at corresponding time points, and was presented as a value relative to body mass (W·kg^−^¹).

For DJ analyses, ground-contact time (DJ-GCT), which was defined as the time subjects were in contact with the ground immediately preceding each jump, was calculated as the time between initial landing and take-off. Take-off and landing was identified as the point when the vertical ground reaction force descended and ascended past 20 N, respectively. Flight time (DJ-FT) was calculated as the time between the points of take-off and landing. Drop-jump RSI was calculated via the alternative method as: RSI = FT/GCT. The mean performance of the three trials for both CMJ and DJ was used for further analysis.

### 2.4. Isometric Mid-Thigh Pull

Isometric strength was assessed during isometric mid-thigh pull (IMTP) testing, using a portable force platform sampling at 600 Hz (400 Series Performance Force Plate; Fitness Technology, Adelaide, Australia) [34]. For the IMTP, subjects obtained self-selected knee and hip angles (knee = 130–150°; hip = 140–160°) based on the reports of previous research [35]. For this test, an immovable, collarless steel bar was positioned at approximately mid-thigh, just below the crease of the hip, using a portable IMTP rig (Fitness Technology, Adelaide, Australia). Each athlete was provided two warm-up pulls, one at 50% and one at 75% of the subjects perceived maximum effort, separated by one minute of rest. Subjects performed three maximal IMTP, with the instruction to pull against the bar with maximal effort as quickly as possible, and push the feet down into the force plate; this instruction has been previously found to produce optimal testing results [36]. Each maximal isometric trial was performed for five seconds, and all subjects were given strong verbal encouragement during each trial. Two minutes of rest was given between the maximal effort pulls.

The peak force recorded from the force-time curve during the five second IMTP trial was reported as the PF, and was presented as a value relative to body mass (N∙kg^−1^). The mean performance of the three was used for further analysis.

### 2.5. Statistical Analysis

Data are presented as either mean ± *SD* or mean with 90% confidence intervals, where specified. Within-session reliability of the variables was examined using the intraclass correlation coefficient (ICC) and coefficient of variation (CV). To assess the magnitude of the ICC, the threshold values were 0.1, 0.3, 0.5, 0.7, 0.9, and 1.0 for low, moderate, high, very high, nearly perfect, and perfect, respectively [37]. Coefficient of variation was calculated as (CV% = *SD*/mean × 100). Normality of data were assessed by Shapiro–Wilk statistic, and homogeneity of variance was verified with the Levene’s test. Independent sample *t*-tests were used for normally distributed data to assess differences CMJ and DJ force-time characteristics between the top 50% [stronger (*n* = 8)] and bottom 50% [weaker (*n* = 8)] with respect to IMTP PF (N·kg^−^¹). Otherwise, Wilcoxon signed ranks tests were used for data that did not meet the assumption of normality. To avoid Type 1 error, a Holm–Bonferroni sequential adjustment was applied as multiple separate comparisons were completed [38]. The magnitude of differences between stronger and weaker players was also expressed as standardized mean difference [Cohen’s *d* effect sizes, (ES)] [39] and interpreted using the scale presented by Hopkins et al. [40]. The threshold for a change to be considered practically important (the smallest worthwhile change [SWC]) was set at 0.2 × between subject SD, based on Cohen’s *d* ES principle. The probability that the magnitude of change was greater than the SWD was rated as <0.5%; *almost certainly not*, 0.5–5%; *very unlikely*, 5–25%; *unlikely*, 25–75%; *possibly*, 75–95%; *likely*, 95–99.5%; *very likely*, and >99.5% *almost certainly* [41]. The effect was deemed unclear when the confidence interval spanned both substantial positive and substantial negative values (±0.2 × between subject SD). All data were statistically analysed using SPSS software (version 17.0, SPSS Inc., Chicago, IL, USA). An a priori alpha level of *p* ≤ 0.05 was used as the criterion for statistical significance.

## 3. Results

**Figure 1:**
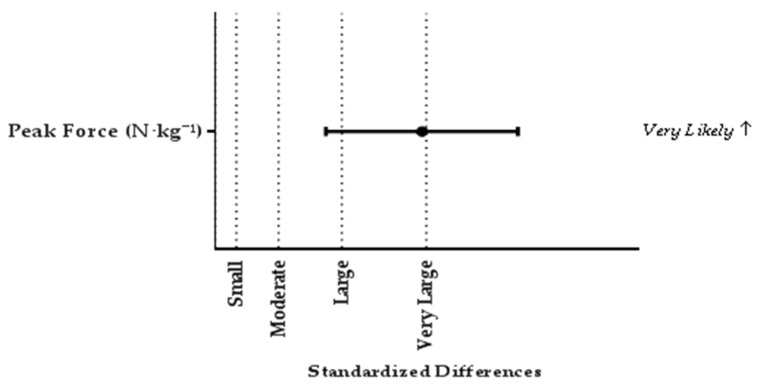
Comparison of IMTP force-time charactericts between stronger and weaker players. Standardized difference was interpreted as: trivial <0.2, small 0.2–0.6, moderate 0.6–1.2, large 1.2–2.0, very large >2.0. Results presented as mean ± 90% confidence limits.

Table 1 shows the IMTP, CMJ and DJ force-time characteristics for stronger and weaker players. Intraclass correlation coefficients and CV demonstrated high to nearly perfect within-session reliability for all IMTP, CMJ and DJ variables (Table 1). Stronger players demonstrated *very likely* differences (*d* = 1.96) in IMTP PF (N·kg^−^¹) compared to weaker players (Figure 1).

**Figure 2:**
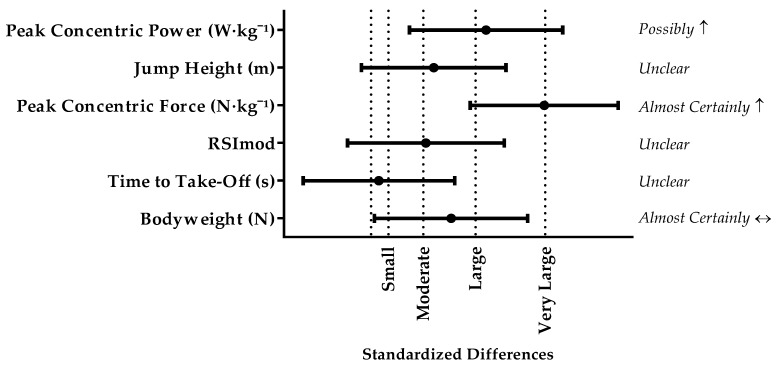
Comparison of CMJ force-time charactericts between stronger and weaker players. Standardized difference was interpreted as: trivial <0.2, small 0.2–0.6, moderate 0.6–1.2, large 1.2–2.0, very large >2.0. Results presented as mean ± 90% confidence limits.

Differences in CMJ-PF demonstrated *almost certain* increases, and there was a *possible* increase in CMJ-PP between stronger and weaker players. There were *unclear* differences in time to take-off, RSImod, and CMJ-JH between groups. The standardised differences between stronger and weaker players ranged from trivial to large (Figure 2).

**Figure 3:**
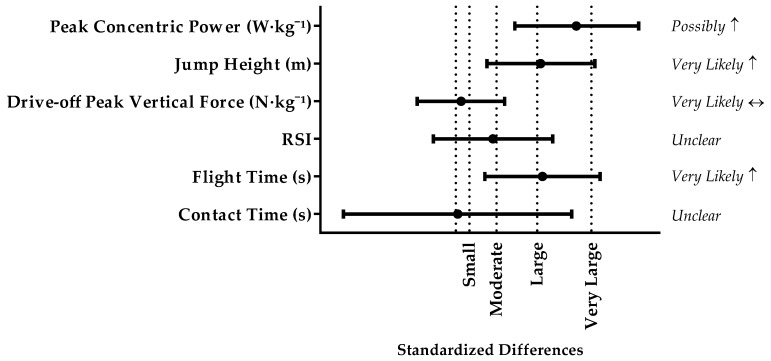
Comparison of DJ force-time charactericts between stronger and weaker players. Standardized difference was interpreted as: trivial <0.2, small 0.2–0.6, moderate 0.6–1.2, large 1.2–2.0, very large >2.0. Results presented as mean ± 90% confidence limits.

There were *very likely* increases in flight time and DJ-JH between stronger and weaker players. Stronger players demonstrated a *possible* increase in DJ-PP (*p* = 0.052; *d* = 1.78) than weaker players. There were *very likely* trivial differences in DJ-PF and *unclear* differences in DJ-GCT, and DJ-RSI between stronger and weaker players. The standardised differences between stronger and weaker players ranged from trivial to large (Figure 3).

## 4. Discussion

The purpose of this study was to explore differences in CMJ and DJ force-time characteristics between stronger and weaker adolescent male basketball players. This study progressed on previous research examining the influence of maximum strength on CMJ [42] and DJ [14,27] performance. There were differences in CMJ-PF between stronger and weaker players. Also, stronger players demonstrated superior DJ-PP than weaker players. However, this study has been unable to find differences in CMJ-JH and DJ-JH between stronger and weaker players. Also, in contrast to our hypothesis, no differences were found between stronger and weaker players in DJ-PF. Differences in force-time characteristics between these players can inform the training and monitoring practices required to improve slow (CMJ) and fast (DJ) SSC abilities of adolescent male basketball players. As expected, stronger players had greater IMTP PF than weaker athletes (strong = 31.75 ± 3.67 N·kg^−^¹; weak = 25.45 ± 2.69 N·kg^−^¹). Increased relative strength would be advantageous in basketball where jumping involves high levels of force production and absorption to overcome the inertia of body mass. Thus, previous studies have confirmed maximum strength to be strongly associated with CMJ force-time measures [1,42,43,44], likely due to similarities in vertically directed force production and the subsequent biomechanically derived variables.

This study found no differences between stronger and weaker players in CMJ-JH. Our findings are in agreement with some [16,42], but in contrast to those who observed differences CMJ-JH between stronger and weaker netball [45] and team-sport athletes [43]. These discrepancies may be explained by methods used to calculate CMJ-JH, sample population and statistical approaches, thus resulting in contradictory findings. Also, the possible interference of sport-specificity cannot be ruled out. For example, basketball athletes may develop familiarity to performing CMJ as it is performed so commonly in training and competition, which may have masked any differences between stronger and weaker players. The finding that CMJ-PF was different between stronger and weaker players is in contrast to previous work [42]. In our study, stronger and weaker players demonstrated similar times to take-off and depth of countermovement, thus these findings cannot be attributed to differences in jump strategy. Therefore, it is likely that players of superior isometric strength levels are better able to apply force during the CMJ. These results further support the idea that maximum strength is strongly associated with CMJ-PF [44]. Lack of differences in CMJ-JH may be attributed to similarities in time to take-off and velocity at take-off. These results may be explained by the fact that basketball athletes regularly perform CMJ’s as part of training and competition, potentially masking any influence of maximum strength on the resultant outputs. Another possible explanation for this is that maximum isometric strength may not play as big a part as first expected when athletes are exposed to lower eccentric loads (slow SSC).

The finding that DJ-RSI, did not discriminate between stronger and weaker players is in line with recent work [14], but in contrast to work in female rugby players [16]. The reason for similar DJ-RSI values demonstrated by stronger and weaker players is perhaps reflective of the *unclear* differences in GCTs produced by both groups. These results are likely to be related to large confidence intervals associated with the standardized difference, which may have been influenced by the small sample size adopted within this study. Likewise, recent research has found no differences between stronger and weaker players in DJ-GCT at various drop heights [14,16]. It should be noted that Barr and Nolte [16] only provided comparisons between groups when combining jump parameters between all drop heights, potentially masking any differences. Given that subjects in the current study were instructed to jump “as high and as fast as possible”, it is unknown whether the results could have differed had subjects been instructed to primarily minimize GCT [29].

This study found no difference in DJ-JH between stronger and weaker players. This outcome is similar to that of Beattie et al. [14] who found no differences between stronger and weaker players in JH at 0.3 m drop height. Also, Barr and Nolte [16] found no differences between stronger and weaker players in DJ-JH from 0.24 and 0.36 m drop heights. As velocity is defined as displacement divided by time, the present results suggest that stronger players achieved similar changes in vertical velocity as weaker players within similar GCTs. As DJ-PP is defined as the product of vertical velocity and vertical ground reaction force, the present results show that both groups exhibited similar DJ-PF and vertical velocities. Though not reported in the current study, there were moderate (*p* = 0.088; *d* = 0.92) differences in body mass between stronger (76.6 ± 7.8 kg) and weaker (85.6 ± 11.6 kg) subjects. Therefore, lack of differences in vertical velocity may be attributed to greater variation in body mass both within and between groups, given that velocity is related to change in momentum (mass × velocity).

Some limitations exist in the current study. First, the current study had a low sample size to compare stronger and weaker players; however, 15 players has previously been representative of a basketball cohort [10,11,46]. Moreover, the size of a confidence interval is influenced by sample size; therefore, the unclear results observed in this study may represent that larger sample sizes are required to understand the certainty of specific CMJ and DJ force-time variables in this subject cohort. Secondly, previous research indicates that vertical jump height is only slightly modified with different countermovement depths [47]; therefore, it seems probable that the RSImod could have been affected by the magnitude of the countermovement (i.e., higher RSImod would be obtained with lower countermovement depth because the time to take-off would be considerably lower, but jump height would remain practically similar). Finally, this study only analyzed DJ force-time characteristics from 0.3 m. Previous research has indicated that performing DJ from incremental drop heights may provide different values of DJ-GCT, DJ-RSI and DJ-JH as compared with 0.3 m. However, this drop height has previously been used to assess reactive-strength in basketball players [46]. Therefore, further research should be undertaken to investigate the role of maximum strength on DJ force-time characteristics in adolescent male basketball players. Such studies may help direct the creation of athlete training and monitoring programs more effectively to represent accurate player profiling, as DJ performed from one drop height may not necessarily predict performance in another.

## 5. Conclusions

This study has shown that relatively stronger players demonstrated superior levels of CMJ-PF, likely at the beginning of the concentric phase, compared to relatively weaker players. The larger CMJ-PF achieved by stronger players was likely attributed to them demonstrating the ability to transfer superior isometric strength to concentric force during the CMJ. However, no differences in DJ force-time characteristics existed between stronger and weaker players. Future work in this area should focus on DJ force-time characteristics from incremental drop heights in relatively strong and weak athletes to help direct the creation of athlete training and monitoring programs more effectively.

## Figures and Tables

**Table 1:** Strength comparison of IMTP, CMJ, and DJ force-time characteristics.

Variable	Strong (*n* = 8)		Weak (*n* = 8)		*p*	*d*	ICC	%CV
	Mean	SD	Mean	SD				
IMTP								
Peak Force (N·kg^−^¹)	31.75	3.67	25.45	2.69	0.026	1.96	0.91	3.81 (2.40)
CMJ								
Time to Take-Off (s)	0.93	0.16	0.91	0.14	0.858	0.09	0.57	9.12 (7.42)
RSImod	0.38	0.09	0.33	0.04	0.237	0.63	0.70	9.46 (7.18)
Peak Concentric Force (N·kg^−^¹)	25.56	1.66	22.84	1.01	0.013	1.99	0.83	2.33 (2.46)
Jump Height (m)	0.34	0.04	0.30	0.05	0.150	0.72	0.98	1.61 (2.32)
Peak Concentric Power (W·kg^−^¹)	53.70	6.68	46.02	4.74	0.228	1.32	0.97	2.38 (1.25)
DJ								
Contact Time (s)	0.38	0.10	0.38	0.08	0.978	0.03	0.91	5.15 (4.59)
Flight Time (s)	0.51	0.04	0.47	0.03	0.228	1.28	0.82	2.56 (1.88)
RSI	1.42	0.28	1.28	0.23	0.289	0.55	0.89	5.05 (4.53)
Peak Concentric Force (N·kg^−^¹)	30.41	6.57	30.85	4.35	0.834	0.08	0.88	5.24 (3.13)
Jump Height (m)	0.32	0.05	0.27	0.03	0.180	1.25	0.84	9.09 (8.05)
Peak Concentric Power (W·kg^−^¹)	55.92	4.27	45.42	7.14	0.052	1.78	0.84	5.30 (4.34)

IMTP = isometric mid-thigh pull; CMJ = countermovement jump; DJ = drop jump; RSImod = reactive strength index-modified; RSI = reactive strength index.
